# Identification, structural characterization, and molecular dynamic simulation of ACE inhibitory peptides in whey hydrolysates from Chinese Rushan cheese by-product

**DOI:** 10.1016/j.fochx.2024.101211

**Published:** 2024-02-10

**Authors:** Guangqiang Wei, Teng Wang, Yiyan Li, Rong He, Aixiang Huang, Xuefeng Wang

**Affiliations:** a College of Food Science & Technology, Yunnan Agricultural University, Kunming 650201, Yunnan, China; b College of Food Science and Engineering, Nanjing University of Finance and Economics, Nanjing 210003, Jiangsu, China

**Keywords:** Rushan cheese whey, ACE inhibitory peptides, LC-MS/MS, Molecular docking, Molecular dynamics simulation

## Abstract

To realize the high-value utilization of Rushan cheese by-product, Rushan cheese whey was used as a raw material to prepare angiotensin-Ⅰ-converting enzyme inhibitory peptides (ACEIPs). After enzymatic hydrolysisn and ultrafiltration, the sequences of peptides were identified by liquid chromatography-mass spectrometry/mass spectrometry (LC-MS/MS). Two novel ACE inhibitory peptides Phe-Asp-Arg-Pro-Phe-Leu (FDRPFL) and Lys-Trp-Glu-Lys-Pro-Phe (KWEKPF) were identified. Additionally, both of the peptides exhibited good water-solubility and no toxicity according to *in-silico* prediction. Fourier transform infrared spectroscopy results show that both FDRPFL and KWEKPF were enriched in *β*-turn and *β*-sheet structures. Lineweaver-Burk plots revealed that FDRPFL and KWEKPF exhibited non-competitive and mixed inhibition patterns, respectively. Molecular docking and MD simulation showed that hydrogen bonds and ionic bonds forces allowed FDRPFL and KWEKPF to form stable and compact complexes with ACE. In conclusion, enzymatic hydrolysis of Rushan cheese by-products yields bioactive peptides, increases the added value of whey and reduces environmental pollution.

## Highlights

• Enzymatic hydrolysis of Rushan cheese by-products yielded bioactive peptides.
• Two novel peptides FDRPFL and KWEKPF with ACE inhibitory activity were identified.
• FDRPFL and KWEKPF showed non-competitive and mixed inhibition patterns, respectively.
• Interaction between the two peptides and ACE was due to hydrogen bonds and ionic bonds.
• MD simulations revealed that two peptides formed stable and compact complexes with ACE.

## Graphical abstract

**Figure:**
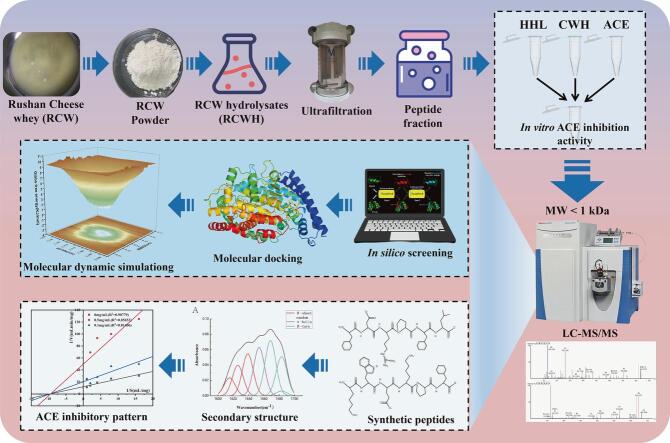

## Introduction

It is well known that excess angiotensin-Ⅰ-converting enzyme (ACE) leads to high blood pressure, and current ACE inhibitors such as captopril, benazepril, enalapril, quinapril and univasc, are widely used in clinical practice for treating hypertension. Nevertheless, the above-described ACE inhibitors can cause adverse effects, such as skin rashes, cough, headaches, fatigue and abnormal taste (Abdelhedi et al., 2018). In contrast, bioactive peptides (BPs) can inhibit ACE and exert a beneficial effect on blood pressure regulation without causing side effects. Hence, antihypertensive peptides derived from natural sources have gained popularity among researchers as a promising alternative for preventing or treating high blood pressure. In particular, enzymatic hydrolysis is an effective method for obtaining BPs through the modification of food proteins due to its high reaction efficiency, ability to yield low harmful by-products, and controllable reaction conditions (Zhao et al., 2022, Zhou et al., 2022).

Milk protein is an important source of BPs whose release from precursor proteins can be achieved via hydrolysis through enzymatic hydrolysis and digestion. Dairy products contain various types of bioactive peptides, including immunomodulatory peptides, ACE inhibitory peptides (ACEIPs) (Cui et al., 2022, Wei et al., 2022), antioxidant peptides (Martini et al., 2020, Martini et al., 2020), antibacterial peptides, and *α*-glucosidase inhibitory peptide (Zhao et al., 2022, Zhou et al., 2022). Acid-coagulated cheese is the typical cheese in the Chinese market, often produced by herdsmen with leftover milk. Rushan cheese, Rubing cheese, Qula, milk tofu, and Kazakh milk bumps are the five traditional acid-coagulated cheese produced in China. In particular, Rushan cheese is a traditional acid-coagulated cheese consumed for over a thousand years in Yunnan province, China, generates 16,000 tons of whey annually during its manufacture process (Wei, Wang, Wang, Shi, & Huang, 2022). Owing to its high mineral and vitamin contents, whey is often used as animal feed. However, the threat of animal salt poisoning and degradation imposes a limitation to its use. Hence, whey has been casually discharged as waste, increasing environmental pollution. In recent years, the development of bioactive peptides from waste has become a research hotspot (Tuly et al., 2022). Therefore, an efficient alternative approach to solving the problem of whey utilization is to employ enzymatic hydrolysis technology to produce whey BPs.

Peptidomics and bioinformatics-based approaches have been successfully employed to screen potential BPs and peptide biomarkers of foods (Martini et al., 2020, Martini et al., 2020). For instance, the ACEIPs SLVYPFPGPI isolated from yak and cow milk hard *chhurpi* cheese was successfully identified using a peptidomics and bioinformatics-based approach (Mma, Rc, Lcp, Sps, & Akra, 2022). Additionally, Coscueta et al. (2022) identified five potential ACEIPs (i.e., SWMHQPP, QSLVYPFTGPIPNSL, YPYQGPIVL, MHQPPQPL and HQPPQPL) in goat casein hydrolysate using *in-silico* methods. Among *in-silico* methods, molecular docking, and molecular dynamics (MD) simulations can be used to screen and characterize the molecular mechanisms of bioactive properties of food-derived peptides. Yu, Guo, Shiuan, Xia, and Liu (2020) reported that ACEIP TNGIIR interacts with the active pockets S1, S2 and S1′ of ACE through hydrogen bonding. Using molecular docking and MD simulations, Yu, Wang, Shuian, Liu, and Zhao (2023) elucidate the interaction mechanism between the immunopeptides VQLSGEEK, GFSGLDGAKG and the receptor Toll-like receptor 4-myeloid differentiation 2 (TLR4-MD2). Furthermore, MD simulations were used to reveal the stable binding of LAPYK and its modified peptides to ACE, and enabled the identification of important residues involved in the binding (Zhang et al., 2022). These *in-silico* methods can reduce the workload of purification of ACEIPs and contribute to the discovery of promising ACEIPs capable of binding to ACE. Therefore, peptidomics and bioinformatics-based approaches provide effective methods for identifying ACEIPs from whey hydrolysates.

In this study, Rushan cheese whey was used as a raw material to prepare ACEIPs. After enzymatic hydrolysis, ultrafiltration separation and sequence identification, two novel ACEIPs were obtained, and the study of their ACE inhibitory activity and structure–activity relation were carried out. Therefore, the findings discussed herein can foster the development of a new bioengineering strategy based on enzymatic hydrolysis of Rushan cheese whey protein to obtain bioactive peptides and increase the added value of whey.

## Materials and methods

### Materials and chemicals

**Supplementary figure 1:**
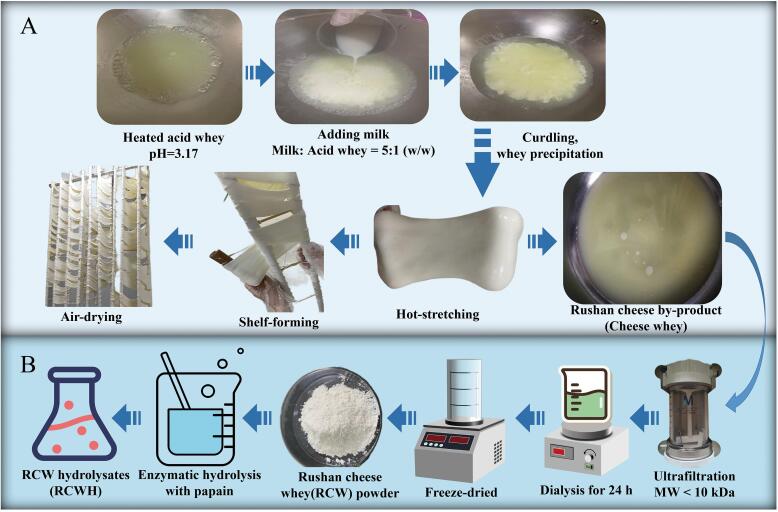


Rushan cheese whey was obtained according to the method described in Fig. S1A. First, 200 mL of naturally fermented acid whey (pH = 3.17) was added to a stainless-steel pan and heated to a boil. Then, 1000 mL of raw milk was transferred to the acidic whey and heated to promote milk gel formation (curdling time: 40 s). The milk gel was collected using a spoon and continuously heat-scalded to obtain a complete Rushan gel. The Rushan gel was submitted to cooking and stretching, then collected in a Rushan holder (cooking-stretching temperature: 70 °C for 50 s). The Rushan gel was dried at room temperature for 18 h to obtain the final Rushan cheese. During this process, whey (a Rushan cheese by-product) was produced.

Papain (100,000 U/g) was purchased from Jiangsu Ruiyang Biotechnology Co. (Jiangsui, China). (*N*-benzoylglycyl)-l-histidyl-l-leucine (HHL) was purchased from Shanghai Yuanye Biotechnology Co. (Shanghai, China). ACE was purchased from Sigma-Aldrich (St. Louis, MO, USA). Amicon Ultra-0.5 mL was purchased from Millipore China Co., Ltd. (Shanghai, China). All other chemicals used were at analytical reagent grade.

### Preparation of whey powder and its hydrolysates

Rushan cheese whey powder was obtained according to the method described in Fig. S1B. Briefly, Rushan cheese whey (300 mL) was ultrafiltered using a stirred UF device (UFSC40001, Millipore, USA) with 80-mm diameter membranes (NMWL, PES, Millipore) and molecular weight cut-off of 10 kDa to obtain fractions with molecular weight below 10 kDa. The ultrafiltered fraction was dialyzed in aqueous solution for 24 h, freeze-dried (Shanghai Bilang Instrument Manufacturing Co., Shanghai, China) and stored in a refrigerator at −20 °C. The composition of obtained whey powder was as follows: protein content, 72.0 %; lactose content, 13.1 % fat content, 9.8 %; moisture content, 4.6 %; total ash content, 8.1 %.

Rushan cheese whey hydrolysates (RCWH) were prepared based on the method of Li et al. (2023) with slight modifications. Briefly, 1 g of whey powder was added to 30 mL of ultrapure water and stirred (IKA Werke GmbH & Co. KG, Staufen, Germany) at 25 °C for 4 h. Then, 0.04 g of papain was added to the mixture and incubated at 50 °C and pH 8.0 at 4 h. After hydrolysis, the enzyme was inactivated by heating at 95 °C for 10 min. The supernatant was obtained by centrifugation (TLG20M, Changsha Maijiasen Instrument Equipment Co., Ltd., China) at 4,000 rfc for 30 min at 4 °C (TLG20M, Changsha Maijiasen Instrument Equipment Co., Ltd., China), then lyophilized and stored at −20 °C.

### Preparation ultra-filtered cut-off fractions of whey hydrolysates

Five different peptide fractions were obtained in the present study, i.e., > 10 kDa, 5–10 kDa, 3–5 kDa and 1–3 kDa, and < 1 kDa, which were separated by using different sequential ultra-filtration membranes (UFSC40001, Millipore, USA) with cut-offs of 10 kDa, 5 kDa, 3 kDa and 1 kDa, respectively. Fractions of whey hydrolysates with different molecular weights (MW) ranges were lyophilized (SCIENTZ-18 N, Bilang Instrument Manufacturing, China) for further peptide identification, and *in-vitro* determination of ACE inhibitory activity.

### Determination of in-vitro ACE inhibitory activity of whey hydrolysate fractions

ACE inhibitory activity of whey hydrolysate fractions with different MW was assessed by a method reported by Cushman and Cheung (1971). In brief, 30 μL of whey hydrolysate fraction was added to 80 μL of 5 mM HHL and incubated at 37 °C for 10 min. Then, 20 μL of 25 mU/ mL ACE was added to the mixture. The reaction was performed at 37 °C for 30 min and then stopped by adding 250 μL of 1 M HCl. Subsequently, 1.7 mL of ethyl acetate was added to the mixture to extract the released hippuric acid. After vigorous stirring for 10 s, the samples were submitted to centrifugation (TLG20M, Changsha Maijiasen Instrument Equipment Co., Ltd., China) at 5,000 rcf at 4 °C for 15 min. Then 1.4 mL of the organic phase was transferred to a fresh test tube. Ethyl acetate was evaporated by placing samples in a boiling water bath. The residue was dissolved in 3 mL of deionized water, and absorbance was measured at 228 nm by enzyme marker (Multiskan FC, Thermo Fisher Scientific). The following formula was used to calculate ACE inhibition rate:

ACE-inhibition rate\%:[1-(C-D)/(A-B)]×100%

Where A represents the absorbance with ACE but without the sample; B the absorbance without ACE and the sample; C the absorbance in the presence of ACE and the sample; and D the absorbance with the sample but without ACE.

### Peptide identification by LC-MS/MS

Whey hydrolysate peptides with MW < 1 kDa fraction were identified using EASY-nLC 1200 (Thermo Fisher Scientific, Waltham, MA, USA) connected to a Q Exactive HF-X mass spectrometer (Thermo Fisher Scientific, Waltham, MA, USA). Prior to the identification, whey hydrolysate samples were desalted using Oasis HLB and Oasis MCX desalting columns (Waters Technology (Shanghai) Co., Shanghai, China). Mobile phases A and B were, respectively, 0.1 % formic acid in water and 0.1 % formic acid in acetonitrile. Samples were separated in an analytical column [C18 column, 75 μm (inner diameter) × 25 cm (column length); Thermo Fisher Scientific, Waltham, MA, USA] at a rate of 300 nL/min. The gradient program used was as follows: (I) 5 % B, 0–29 min; (II) 23 % B, 30–37 min; (III) 29 % B, 37–42 min; (IV) 38 % B, 43–44 min; (V) 48 % B, 45–46 min; (VI) 100 % B, 47–60 min. Subsequently, MS/MS raw files were collected and searched using PEAKS Studio 8.5 (Bioinformatics Solutions Inc. Waterloo, Canada) and the UniProt Protein Database. The scanning range was 400–2000 Da. The first 20 ions were separated by collision-induced dissociation for MS/MS. The fragment mass tolerance was set to 10 ppm and 0.02 Da. A false discovery rate (FDR) of 1 % was adopted to validate the assay. Peptides were considered positive in an RCWH when they were identified in ALL replicates for the three given RCWH samples.

### In-silico analysis of identified peptides

The peptides identified in whey hydrolysate were investigated in relation to bioactive peptides previously identified in the literature using the BIOPEP (https://www.uwm.edu.pl/biochemia/index.php/pl/biopep), EROP-Moscow (https://erop.inbi.ras.ru/) and MBPDB search (https://mbpdb.nws.oregonstate.edu/) databases. Only peptides with 90 % homology to the known functional peptides were considered as bioactive peptides. In addition, determining the probability of a peptide having ACE inhibitory activity was conducted using the PeptideRanker (https://distilldeep.ucd.ie/PeptideRanker/) and PepSite 2 (https://pepsite2.russelllab.org/) servers. Peptides with a score > 0.7 were regarded as putative ACEIPs and were used for further virtual screening, where the higher the score, the higher probabilities of the peptide to be bioactive. The hydrophobicity of peptides was evaluated using the PepDraw server (https://pepcalc.com). The water solubility of peptides was analyzed using Innovagen (https://www.innovagen.com/proteomics-tools). The toxicity of peptides was assessed using ToxinPred (http://crdd.osdd.net/raghava/toxinpred/).

### Solid-phase synthesis of peptides

Based on the results of *in-silico* analysis, five peptides (FDRPFL, YDFYPR, DDFFHR, GKWEKPF and KWEKPF) were synthesized in Anhui Guoping Pharmaceutical Co., LTD (Hefei, Anhui, China) using Fmoc solid-phase method and were stored at −20 °C under desiccation. The purities of the peptides were determined by HPLC (purity ≥ 95 %). In addition, the *in-vitro* ACE inhibitory activity, ACE inhibition pattern, and secondary structure of synthetic peptides were evaluated.

### Determination of peptides secondary structure by Fourier transform infrared spectroscopy (FTIR)

The secondary structures of FDRPFL and KWEKPF were determined in a Nicolet 6700 Fourier infrared spectroscope (Thermo Electron Co., Madison, USA). Briefly, 1 mg of freeze-dried synthetic peptide powder was mixed with 100 mg of potassium bromide, ground for 15–20 min using an onyx mortar and pressed into forming 1–2 mm slices which were held at a pressure of 14 kg for 1 min and immediately placed in the FTIR for scanning. FTIR spectra were obtained within the range of 400–4,000 cm^−1^ with a resolution of 4 cm^−1^ air as the background, and 64 scans were performed at a wave number accuracy of 0.01 cm^−1^. FTIR spectra were analyzed using PeakFit V4.12 software (Systat Software Inc, USA). The content of *β*-sheet, *β*-turn, random coil, and *α*-helix of the peptides was calculated according to the method proposed by Cai and Singh (1999).

### Determination of ACE inhibition pattern

The ACE inhibition patterns of FDRPFL and KWEKPF were further evaluated using Lineweaver-Burk plots. Briefly, ACE inhibitory activity was determined by adding 25 μL of ACE inhibitory peptide solutions at different concentrations (i.e., no inhibitor, 0.5 mg/mL, and 1.0 mg/mL) to the reaction system with varying concentrations of HHL (i.e., 0.3125 mmol/L, 0.625 mmol/L, 1.25 mmol/L, 2.5 mmol/L, and 5 mmol/L). Figures were plotted using 1/S as the x value and 1/V as the y value. The type of inhibition pattern determined the peptide was determined using Lineweaver-Burk double reciprocal plots.

### Molecular docking analysis

Molecular docking analysis was conducted using Autodock Vina v.1.1.2 software. The relevant parameters of the ACE target (PDB ID:1O86) were as follows: center x, 40.79; y, 33.61; and z, 43.48; the remainder parameters were set to default settings. Hydrogen bonds, ionic bond, and hydrophobic interactions between the peptide and ACE were determined using Pymol2.3.0 and LIGPLOT v2.2.4 software (Schrodinger, Inc, USA).

### MD simulation

MD simulations were carried out using the GROMACS 2019.6 software. The simulation box size was optimized considering the distance between each atom of the protein and the box greater than 1.0 nm. The box was filled with water molecules considering density of 1. To achieve electrical neutrality, water molecules were replaced with Cl^-^ and Na^+^ ions. Energy optimization of 5.0 × 10^4^ steps was performed following the steepest descent method to minimize the energy consumption and unreasonable contact or atom overlap in the entire system. Then, first-phase equilibration was performed with the NVT ensemble at 300 K for 100 ps to stabilize system temperature, and the second-phase equilibration was simulated with the NPT ensemble at 1 bar and 100 ps. The primary objective of the simulation was to optimize the interaction among the target protein, the solvent and ions, in order to obtain a fully pre-equilibrated simulation system. All MD simulations were performed at 50 ns under an isothermal and isostatic ensemble at a temperature of 300 K and 1 atm. Temperature and pressure were controlled using V-rescale and Parrinello-Rahman methods, respectively; temperature and pressure coupling constants were 0.1 and 0.5 ps, respectively. Lennard-Jones function was used to calculate Van der Waals forces, and nonbond truncation distance was set to 1.4 nm. Bond length was constrained by the LINCS algorithm. Long-range electrostatic interaction was calculated using the Particle Mesh-Ewald method with Fourier spacing of 0.16 nm. Root-mean-square deviation (RMSD), radius of gyration (Rg), solvent-accessible surface area (SASA), and root mean square fluctuation (RMSF) values were calculated to analyze the stability of the FDRPFL and KWEKPF and ACE complexes.

### Statistical analysis

All the experiments were performed in triplicates (n = 3), and results were expressed as mean ± standard deviation. The differences were tested by one-way ANOVA followed by Duncan’s multiple range test. All statistical analyses were conducted by SPSS software (Version 25, SPSS Inc, Chicago, IL, USA). *P* values < 0.05 indicated statistically significant difference between groups. Origin 2021b (OriginLab Inc., CA, USA) was used to visualize obtained data.

## Results and discussion

### ACE inhibition activity

**Fig. 1:**
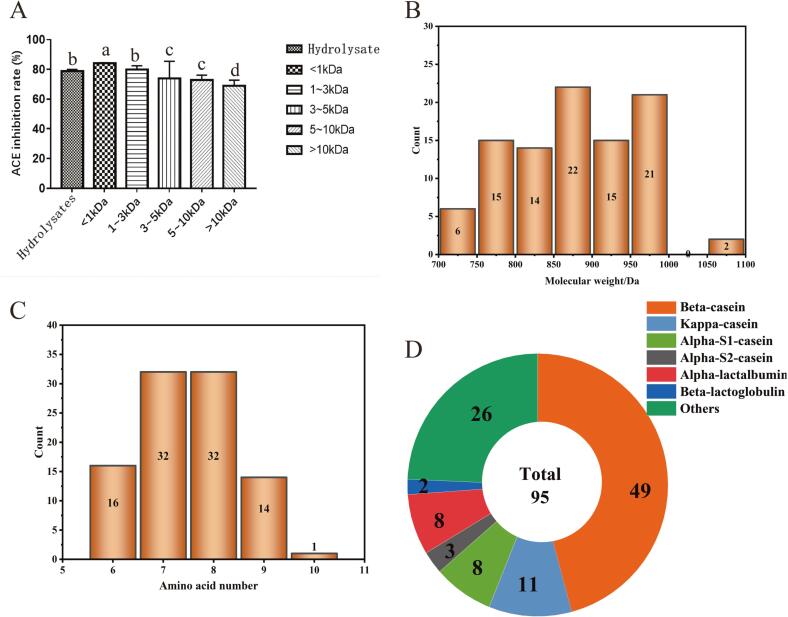
Antihypertensive activity and peptide profile of Rushan cheese whey hydrolysates (RCWH). (A) *In-vitro* angiotensin-Ⅰ-converting enzyme (ACE) inhibitory activity of RCWH. (B) Molecular weight distribution of identified peptides. (C) Amino acid number distribution of identified peptides. (D) Peptides number of identified peptides from beta-casein, kappa-casein, alpha_s2_-casein, alpha_s1_-casein, alpha-lactalbumin, beta-lactoglobulin and other proteins.

The *in-vitro* ACE inhibition activity of RCWH and their ultrafiltered fractions is shown in Fig. 1A. Both RCWH and their ultrafiltered fractions showed ACE inhibitory activity; in particular, the < 1 kDa ultrafiltered fraction demonstrated the highest ACE inhibitory activity (83.6 ± 0.27 %; *P* < 0.05). According to Shih, Chen, Wang, and Hsu (2019) low MW peptides had higher ACE inhibitory capacity *in-vitro*, which corroborated the above results. Previous studies have also shown that whey protein hydrolysates have potential ACE inhibitory activity (Bustamante, Jesús Gil González, Sforza, & Tedeschi, 2021). Cheung, Aluko, Cliff, and Li-Chan (2015) reported that whey protein hydrolysates showed ACE inhibitory activity (> 50 %), and Li et al. (2022) reported that the IC_50_ value of ACE inhibitory activity of whey protein hydrolysate was 1.64 mg/mL. Another study reported that the IC_50_ value for ACE inhibition of *α*-lactalbumin hydrolysate was 0.103 mg/mL (Xie et al., 2022). In addition, dipeptides LL, KA, LF, and EN, as well as tripeptides DIS, EVD, AIV, and VFK with hypotensive activity were identified from whey protein hydrolysates (Pan et al., 2020, Guo et al., 2018). Collectively, these results suggested that the enzymatic hydrolysates of Rushan cheese whey powders may be critical for the release of peptides with potential ACE inhibitory activity. These findings contribute to the development of novel enzymatic-based bioprocesses for the production of ACEIPs from wasted cheese whey.

### Peptide profiling

Liquid chromatography-mass spectrometry/mass spectrometry (LC − MS/MS)-based peptidomics was used to identify peptide composition of the < 1 kDa ultrafiltered fraction of RCWH. A total of 95 peptides were identified from the ultrafiltered fractions and whose MW was within the range of 700–1100 Da, but mainly concentrated within the range of 850–1000 Da (Table S1; Fig. 1B). The analysis of amino acid composition of ultrafiltered fractions showed that the identified peptides contained 6–10 amino acid residues, being mainly composed of 7–8 amino acid residues (Fig. 1C). Interestingly, 53 of the identified peptides contained the amino acid residues Phe (F), Arg (R), and Lys (K) either at the *N*-terminus or C-terminus, which corresponded to the specific cleavage site of papain (Table S1) (Zhang et al., 2017). In addition, most of the identified peptides derived from casein and whey proteins, including *β*-casein 49, *κ*-casein 11, *α_s1_*-casein 8, *α_s2_*-casein 3, *α*-lactalbumin 8, and *β*-lactoglobulin 2 (Fig. 1D). *α*- and *β*-casein are the main components of casein, accounting for 50 % and 40 % of casein, respectively. Moreover, the open structure of these two proteins is easily hydrolyzed by enzymes, such as papain (Jin et al., 2016). During enzymatic hydrolysis, papain mainly hydrolyzes casein, especially *β*-casein, to release peptides. Notably, 11 and 2 peptides originated from *α*-lactalbumin and *β*-lactoglobulin, respectively, suggesting that papain can also hydrolyze whey proteins to release peptides. Interestingly, only a very small number of peptides from whey proteins was identified, likely due to two reasons: i) considering that LC-MS/MS is an ion saturation instrument, and that high abundant proteins affect the identification of low abundant proteins, the high abundance of casein might affect the identification of *α*-lactalbumin and *β*-lactoglobulin (Jin et al., 2016); ii) *α*-lactalbumin and *β*-lactoglobulin were completely hydrolyzed to amino acids or to low-molecular-weight peptides which could not be identified (Shi, Wei, & Huang, 2021).

### Prediction of bioactive peptides

Subsequently, the potential function of identified peptides in RCWH was predicted based on BIOPEP, EROP-Moscow, and MBPDB databases. As shown in Table S2, 13 peptides in the BPs database showed ACE inhibitory, antioxidant, dipeptidyl peptidase IV inhibitory, and antibacterial activities. The results showed that enzymatic hydrolysis of Rushan cheese by-products yields BPs, increases the added value of whey and reduces environmental pollution. Interestingly, most of the identified BPs are derived from *β*-casein, which can be explained by the high and variable content of *β*-casein in casein and whose open structure can be easily enzymatIcally digested. Moreover, ACEIPs (n = 10) were the most abundant potentially BPs in RCWH. Especially, the peptide VYPFPGPI was found to have ACE inhibitory and antioxidant activities in fermented milk, casein and whey hydrolysates (Pihlanto, Virtanen, & Korhonen, 2010). The peptide DKIHPF isolated from milk fermented with *Lactobacillus delbrueckii* subsp. *bulgaricus* SS1 and *Lactococcus lactis* subsp. *cremoris* FT4 was shown to have ACE inhibitory activity (Gobbetti, Ferranti, Smacchi, Goffredi, & Addeo, 2000). In addition, the peptide DKVGINYW derived from *α*-lactalbumin showed ACE inhibitory activity and was identified in whey protein Ioncentrate (WPC) hydrolysates (Tavares & Malcata, 2012). In general, peptides with ACE inhibitory activity have hydrophobic amino acids residues, including Tyr (Y), Phe (F), Pro (P), Trp (W) at the C-terminus (Wei et al., 2022). In the current study, the potential ACEIPs YPFPGPIP, VYPFPGPIP, DKIHPF, DKVGINYW and DKVGINY all contained hydrophobic amino acids residues at the C-terminus. The potential ACEIPs in RCWH contributed to ACE inhibitory activity, which is consistent with the results of *in-vitro* experiments. Collectively, these results suggest that papain mainly hydrolyzed *β*-casein to release BPs, especially ACEIPs. In particular, papain hydrolyzed peptide bonds L58–V59 and I66–H67 of *β*-casein to release ACEIPs and the antioxidant bifunctional peptide YPFPGPI; hydrolyzed peptide bonds Q167-–168 and Q175–K176, Q182R183 to release ACEIPs SKVLPVPQ and KVLPVPQK; hydrolyzed peptide bonds L115–D116 and W123–L124 of *α*-lactalbumin to release the ACEIP DKVGINYW; and hydrolyzed peptide bonds E61–L62 and E71–I72 of *β*-lactoglobulin to release the ACEIP LKPTPEGDLE.

### In-vitro and in-silico strategy analyses of novel ACE inhibitory peptide

Peptidomics results indicated that RCWH is an important source of ACEIPs (Bustamante, Jesús Gil González, Sforza, & Tedeschi, 2021). Subsequently, a screening for novel potential ACEIPs isolated from RCWH was conducted using an *in-vitro* and *in-silico* strategy. As shown in Table 1, five novel potential ACEIPs were screened based on the *in-silico* strategy. PeptideRanker is an online platform that establishes a score for the biological activity of peptides based on certain characteristics such as amino acid composition, length, and hydrophobicity (Mudgil et al., 2019). The higher the score, the higher the probability that the peptide is biologically active. In the current work, five peptides with PeptideRanker scores > 0.70 were considered to possess potential ACE inhibitory activity. Furthermore, we then analyzed the *P*-values of these five peptides interacting with ACE using the computational prediction software Pepsite2, enabling rapid screening of potential ACEIPs. The Pepsite2 prediction results showed that the five novel ACEIPs showed potent interaction with ACE as revealed using Pepsite 2 with *P* < 0.05 (Mudgil et al., 2019). The peptide FDRPFL had the lowest *P*-value (*P* = 0.0001171), followed by KWEKPF (*P* = 0.0001704), which indicated that FDRPFL and KWEKPF could bind more strongly to ACE compared to the other three peptides. Pepsite 2 predictions suggested that FDRPFL and KWEKPF exhibit stronger ACE inhibitory activity compared to the other three peptides. In addition, predictions of physicochemical properties showed that the five low-MW peptides (MW < 1000 Da) exhibited good water solubility, high hydrophobicity and non-toxicity.

**Table 1:** Biochemical properties and angiotensin-I-converting enzyme (ACE) inhibition rate (IC_50_ values) of five novel peptides obtained from Rushan cheese whey hydrolysates.

Peptide sequences	Length	Molecular weight/Da	Peptide ranker score	PepSite 2P value	Solubility	Toxicity	IC_50_ (mg/mL)	Hydrophobicity/%
FDRPFL	6	793.4122	0.973047	0.0001171	Good	Non-toxic	0.67 ± 0.04	66.67
YDFYPR	6	859.3864	0.870709	0.0002972	Good	Non-toxic	>1	33.33
DDFFHR	6	835.3613	0.867097	0.0005947	Good	Non-toxic	>1	33.33
GKWEKPF	7	890.465	0.79245	0.0004013	Good	Non-toxic	>1	42.86
KWEKPF	6	833.4435	0.764034	0.0001704	Good	Non-toxic	0.75 ± 0.11	50.00

**Supplementary figure 2:**
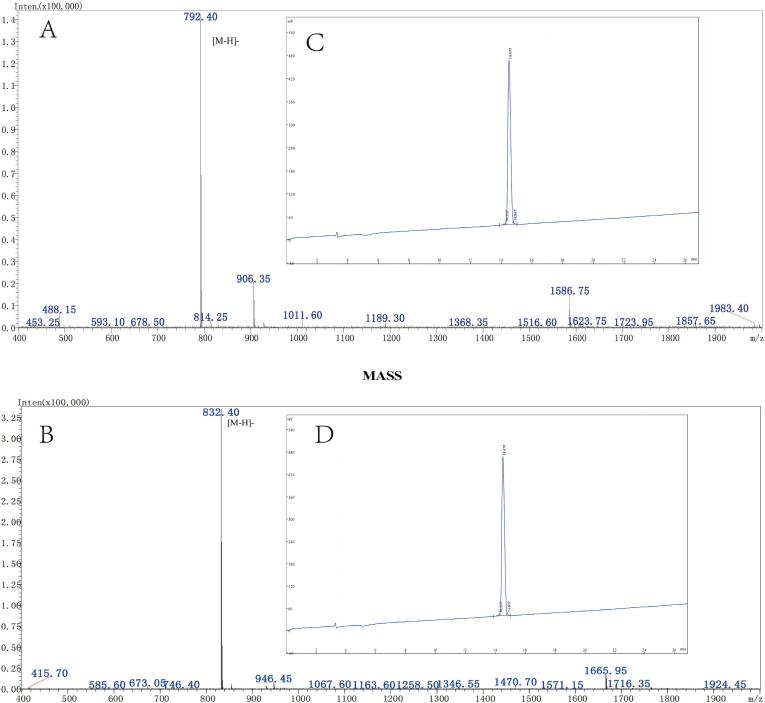


The IC_50_ values were defined as the concentration of the inhibitor required to inhibit 50 % of the ACE activity. Subsequently, the five novel peptides were synthesized and their IC_50_ values for ACE inhibitory activity were determined, and whose results are shown in Table 1 and Fig. S2; only the liquid phase diagram of FDRPFL and KWEKPF is shown. Table 1 depicts the IC_50_ value of the five synthetic peptides as calculated according to regression equations. Among these, FDRPFL showed the highest ACE inhibitory activity with an IC_50_ value of 0.67 ± 0.04 mg/mL, followed by KWEKPF with an IC_50_ value of 0.75 ± 0.11 mg/mL. Noteworthy, the IC_50_ values of the ACE inhibitory activity of FDRPFL and KWEKPF were lower than those of peptides derived from eel bone (i.e., GPIGPPGPR, PMGPR, GPMGPR, GPAGPR, GPSGAPGPR and GGPGPSGPR with respective IC_50_ values of 1.441, 1.47, 2.247, 1.246, 1.022 and 0.758 mg/mL), dipeptide GA (1.22 mg/mL) purified from whey proteins resulting from fermentation using *Lactobacillus plantarum* QS670, and *α*-lactalbumin derived from ACE inhibitory peptides PEW (3,130 μmol/L) (Shao, 2022, Xia et al., 2020, Hou et al., 2021). However, IC_50_ values of the ACE inhibitory activity of FDRPFL and KWEKPF were slightly higher than that of LLAGGW (IC_50_ = 0.49 mg/mL) and LGYSF (IC_50_ = 0.51 mg/mL) which were derived from large yellow croaker (Li, 2021).

It is known that the biological activity of a peptide is closely related with its amino acid sequence. In particular, the presence of aromatic (Phe (F), Try (W) and Tyr (Y)) and hydrophobic (Ala (A), Val (V), Leu (L), Pro (P) and Glu (E)) amino acid residues is associated with ACE inhibitory activity of BPs (Sanjukta et al., 2021). In addition, the presence of Pro (P) residue increases the ACE inhibitory activity of the peptide. As shown in Table 1, the hydrophobicity of FDRPFL was 66.67 %. Thus, the C-terminal hydrophobic amino acid residues Leu (L) and the *N*-terminal hydrophobic amino acid residue Phe (F) in FDRPFL likely contributed to its ACE inhibitory activity. Moreover, the hydrophobic content of KWEKPF was 50.00 %, which was lower only than that of FDRPFL, and consequently the ACE inhibitory activity of KWEKPF was lower than that of FDRPFL. In contrast, the synthetic peptides YDFYPR, DDFFHR, and GKWEKPF showed low ACE inhibitory activity, with IC_50_ values greater than 1 mg/mL. This indicates that the activity of BPs is not only related to their amino acid composition but also to peptide conformation. This work proposed an *in vitro*-*in silico* strategy for the rapid discovery of ACIPs. The RCWH derived ACEIPs possess good processing properties and safety, which are important to the application of these peptides in food industry. These findings suggest that RCWH has a potential to be the source of natural ACEIPs.

### Structural characterization of ACE inhibitory peptide

#### Primary and secondary structure of ACE inhibitory peptide FDRPFL and KWEKPF

**Fig. 2:**
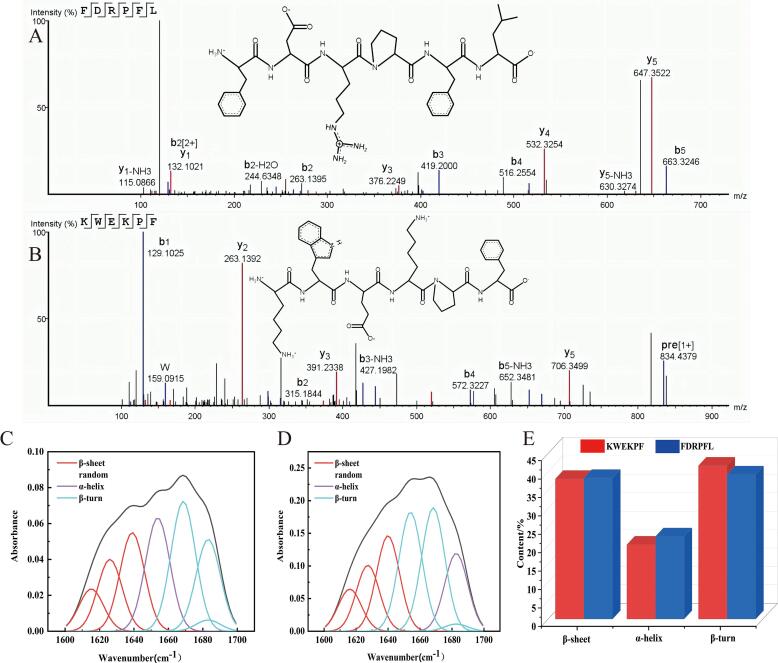
Primary and secondary structure of peptides FDRPFL and KWEKPF obtained from Rushan cheese whey hydrolysates (RCWH). Molecular mass and amino acid sequence of peptides (A) FDRPFL and (B) KWEKPF. Secondary structure spectra of peptides (C) FDRPFL and (D) KWEKPF. (E) Secondary structure composition of peptides FDRPFL and KWEKPF.

The primary structures of FDRPFL and KWEKPF are shown in Figs. 2A and 2B, respectively. The molecular masses of peptides Phe-Asp-Arg-Pro-Phe-Leu (FDRPFL) and Lys-Trp-Glu-Lys-Pro-Phe (KWEKPF) were 793.4122 and 833.4435 Da, respectively, and both consisted of six amino acids residues. The secondary structure of a polypeptide refers to the local arrangement of its amino acid residues, which interact by forming hydrogen-bonding and van der Waals forces. The secondary structure of peptides has been reported to play a potential role in determining ACE inhibitory activity (Yu et al., 2012). Fig. 2C–E depicts secondary structure spectra and contents of FDRPFL and KWEKPF. As shown in Fig. 2E, FDRPFL was composed of 39.26 % *β*-turns, 38.32 % *β*-sheets and 22.43 % *α*-helices; KWEKPF was composed of 41.66 % *β*-turns, 38.05 % *β*-sheets and 20.20 % *α*-helices. These findings collectively suggested that the relative amounts of *β*-turns and *β*-sheets were the main contributors to the ACEI inhibitory activity of these two peptides, which was consistent with the findings of Tu et al. (2018) who reported that *β*-sheets in the peptide NMAINPSKENLCSTFCK played an important role in its ACE inhibitory activity. Moreover, it was found that the usual peptide secondary structure with a greater number of hydrogen bonds in *β*-turn corners was a more ordered and regular structure, thus indicating that peptides FDRPFL and KWEKPF had a stable structure (Tanzadehpanah et al., 2016).

#### Mechanism of action of ACE inhibitory peptide FDRPFL and KWEKPF

**Fig. 3:**
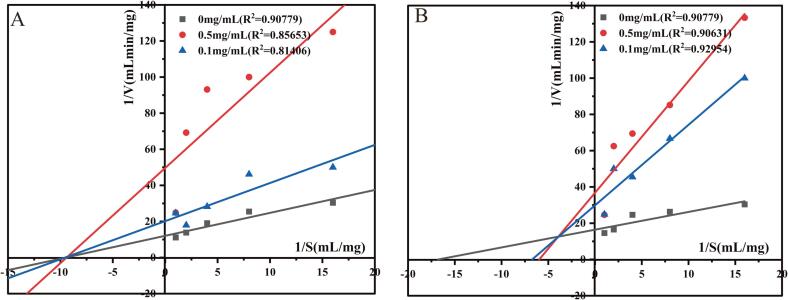
Lineweaver-Burk plots with the angiotensin-Ⅰ-converting enzyme (ACE) inhibition pattern of peptides (A) FDRPFL and (B) KWEKPF at different concentrations.

There are four mechanisms of enzyme inhibition, i.e., competitive, uncompetitive, non-competitive, and mixed modes. The two novel peptides FDRPFL and KWEKPF with high ACE inhibitory activity were further evaluated to confirm their inhibitory mechanism. Fig. 3 depicts Lineweaver-Burk plots with the ACE inhibition pattern of synthetic peptides FDRPFL and KWEKPF at different concentrations. FDRPFL had a similar reaction constant (Km) to that of unblocked enzyme, which indicated that FDRPFL was a non-competitive ACE inhibitor which binds to the inactive site of ACE and does not affect the binding of the substrate to ACE; therefore, the inhibition pattern of FDRPFL onto ACE occurs by forming a ternary complex with the ACE-substrate (Fig. 3A). Several food-derived non-competitive ACEIPs have been described, including GVSLPEW, GYGGVSLPEW and VGINYW from alpha-lactalbumin, KIGSRSRFDVT from shiitake mushroom (Xie et al., 2022, Paisansak et al., 2021).

In contrast to FDRPFL, KWEKPF at different concentrations showed different y-intercepts with lower Vmax and higher Km values, which suggested that KWEKPF established a mixed competitive inhibition pattern (Fig. 3B), and that it could interact with both the active and non-active sites of ACE. Mixed competitive inhibition is typically suggested by a decrease in Vmax and an increase in Km values. The decrease in Vmax values was likely due to the capacity of KWEKPF to bind to the allosteric site of ACE noncompetitively and then alter protease conformation, whereas the increase in Km was likely due to the interaction between KWEKPF and the active site of ACE competing for the substrate. Several ACEIPs with mixed inhibitory patterns have been reported in recent years, including LSGYGP derived from tilapia (Chen et al., 2020), SLPEW and VSLPEW derived from alpha-lactalbumin (Xie et al., 2022).

### Molecular docking analysis

**Fig. 4:**
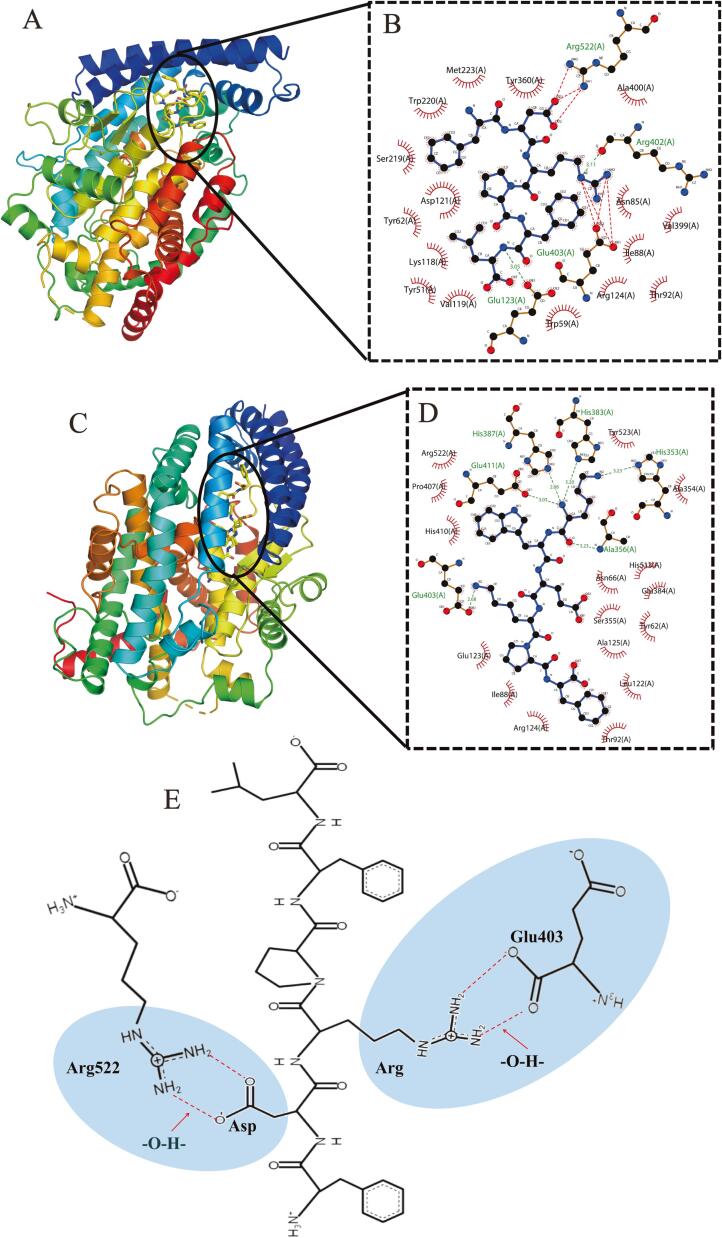
Molecular docking results of peptides FDRPFL and KWEKPF to angiotensin-Ⅰ-converting enzyme (ACE; PDB ID: 1O8A). Three-dimensional binding mode of peptides (A) FDRPFL and (C) KWEKPF to ACE. Interaction force model of (B) FDRPFL and (D) KWEKPF to the ACE pocket. (E) Diagram of the binding mechanism of FDRPFL to ACE via ionic bonding.

Molecular docking methods are often used to explore the interaction of ligands (peptides) and target receptors (enzymes) at the atomic level to identify potential BPs that do not require peptide synthesis and the subsequent analysis of their activity (Zhao et al., 2022, Wei et al., 2022). To explore the molecular mechanism of interaction between ACE and FDRPFL or KWEKPF, molecular docking simulation analysis was conducted using PyMOL, and the model and respective results are shown in Fig. 4 and Table S3. As shown in Table S3, the binding energies of FDRPFL and KWEKPF to ACE were −9.60 and −7.40 kcal/mol, respectively, indicating that both peptides could bind to ACE. Furthermore, molecular docking analysis revealed that FDRPFL and ACE interacted via hydrogen bonds, ionic bonds and hydrophobic forces (Fig. 4B). In particular, amino acid residues Arg (R) and Leu (L) in FDRPFL formed hydrogen bonds with Glu123 and Arg402 of ACE with bond lengths of 3.05 Å and 3.11 Å, respectively. In addition, FDRPFL established hydrophobic interactions with residues Trp59, Arg124, Thr92, Ile88, Val399, Asn85, Ala400, Tyr360, Met223, Trp220, Ser219, Asp121, Tyr62, Lys118, Tyr51, and Val119 of ACE, as well as formed ionic bonds with residues Glu403 and Arg522 (Fig. 4A-B).

Similarly, the amino acid residue Lys (K) in KWEKPF was shown to establish six hydrogen bonds with residues Glu403, Glu411, His387, His383, His353 and Ala356 of ACE with hydrogen bond lengths of 2.68 Å, 3.03 Å, 2.68 Å, 3.20 Å, 3.23 Å and 3.23 Å, respectively (Fig. 4C-D). The ACEIP LYQEPVLGPVR isolated from yak and cow milk hard chhurpi cheese was shown to form hydrogen bonds with amino acid residues Glu411 and Tyr523 of ACE (Mma et al., 2022). In addition, both peptides were shown to interact hydrophobically with 16 amino acid residues of ACE. Previous studies have shown that hydrophobic bonds are not a major force in interactions between peptides and ACE (Mirzaei, Mirdamadi, Ehsani, & Aminlari, 2018). Peptides primarily exert ACE inhibitory activity by binding to the ACE active site which has three pockets, namely S1, S2 and S′1. The S1 pocket includes residues Ala354, Glu384 and Tyr523, the S2 pocket includes residues Gln281, His353, Lys511, His513 and Tyr520, and the S′1 pocket includes a Glu 162 residue. KWEKPF formed six hydrogen bonds with amino acid residues in ACE, one of which (His353) was located in the S2 pocket, which suggests that His353 in the active site of ACE contributes to strengthening the binding between KWEKPF and ACE, and it was consistent with the results of mixed competitive mode of ACE inhibition pattern. Interestingly, FDRPFL did not bind to the active site of ACE, but showed higher ACE inhibitory activity compared to KWEKPF, and likely related to the ACE inhibition pattern of FDRPFL, which was non-competitive, thus indicating that FDRPFL could interact with the inactive site of ACE and its ACE inhibitory activity. Moreover, it was observed that seven ionic bonds were formed between FDRPFL and ACE. As shown in Figs. 4B and 4E, oxygen ions (O^-^) in Asp of FDRPFL formed an O—H ionic bond with the hydrogen ion (H^+^) in residue Arg22 of ACE. Similarly, the hydrogen ion (H^+^) in Arg of FDRPFL formed an O—H ionic bond with oxygen ions (O^-^) in residue Glu403 of ACE. It is well known that ionic bonding forces are stronger than hydrogen bonding forces for interaction forces formed between peptides and ACE. The formed ionic bonds (O—H) contributed to establishing electrostatic force interactions which increased the binding energy of FDRPFL and ACE, which could explain the high ACE inhibitory activity of FDRPFL.

### MD simulations of ACE-ACEIPs complexes

**Fig. 5:**
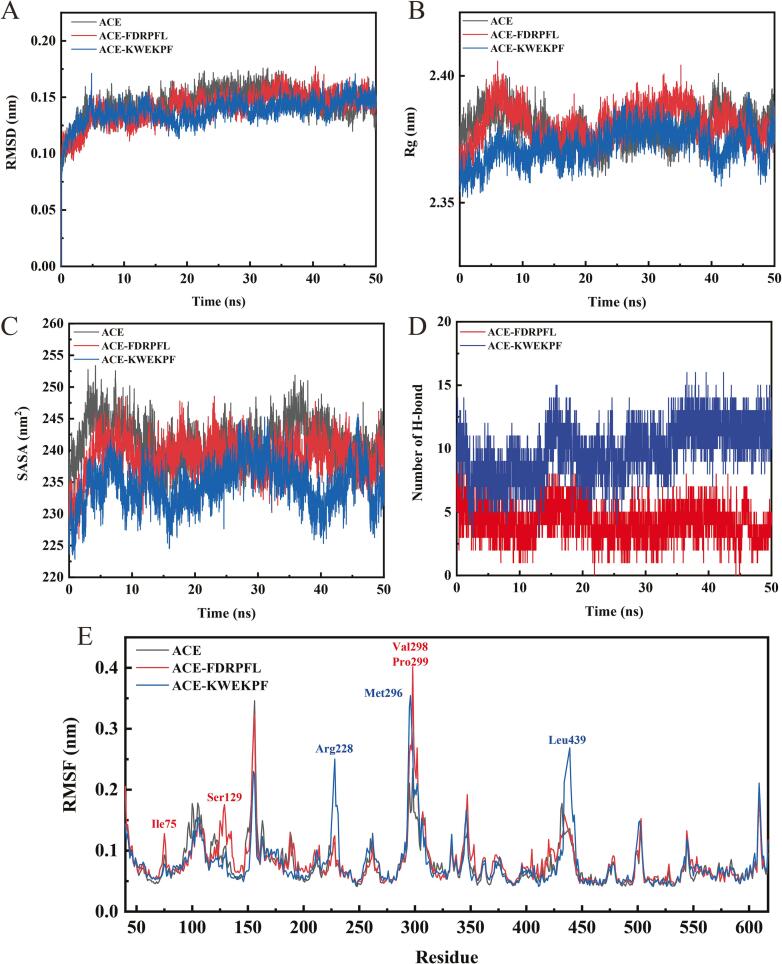
Molecular dynamics simulations (50 ns) of complexes formed between angiotensin-Ⅰ-converting enzyme (ACE) and peptides FDRPFL and KWEKPF. (A) Root mean square deviations (RMSD) (nm). (B) Radius of gyration (Rg) of backbone atoms (nm). (C) Solvent accessible surface area (SASA) values (nm^2^). (D) Number of hydrogen bonds. (E) Root mean square fluctuation (RMSF) of ACE backbones for complexes (nm).

Molecular docking results indicated that ionic and hydrogen bonding forces promote the binding of FDRPFL and KWEKPF to ACE. Thus, to further investigate the molecular mechanisms underlying the stability of the ACE–peptide complexes, the interactions occurring in complexes ACE–FDRPFL and ACE–KWEKPF were studied using MD simulations (50 ns). The binding trajectories of FDRPFL and KWEKPF with ACE were revealed by calculating RMSD, Rg, RMSF, and SASA (Fig. 5).

#### Analysis of RMSD values

RMSD denotes conformational fluctuations in the molecular structure of a peptide at each moment compared to the initial structure. As shown in Fig. 5A, RMSD values of ACE–FDRPFL and ACE–KWEKPF complexes initially increased and then stabilized; mean RMSD values of ACE–FDRPFL and ACE–KWEKPF complexes were 0.1410 and 0.1316 nm, respectively, which were lower than that of ACE (0.1445 nm), thus indicating a change in ACE structure. Moreover, molecular docking results showed that ligands FDRPFL and KWEKPF affected the ACE receptor, since FDRPFL and KWEKPF occupied more active pockets of ACE and established a greater number of interaction forces, which led to traction of ACE active residues and induced conformational changes (Fig. 4B and 4D). Taken together, MD simulation results indicated that both FDRPFL and KWEKPF can interact with ACE, and the formed complexes reached a relatively stable state within 50 ns of simulation.

#### Analysis of Rg and SASA values

In MD simulations, Rg and SASA values can be used to determine the compactness of a protein-peptide complex. In general, lower Rg and SASA values indicate a complex with a more compact molecular structure. Rg values of the ACE–KWEKPF complex fluctuated less during MD simulations, whereas Rg values of non-complexed ACE and the ACE–FDRPFL complex fluctuated more (Fig. 5B). Consistent with the trend observed for Rg values, SASA values for ACE–FDRPFL and ACE–KWEKPF complexes were generally lower than those for non-complexed ACE in MD simulations, suggesting that the binding of FDRPFL and KWEKPF to ACE resulted in less structural changes, thus demonstrating the stability of the protein-peptide complex (Fig. 5C).

#### Analysis of RMSF values

Fig. 5D shows the number of hydrogen bonds formed between ACE and FDRPFL or KWEKPF over a simulation time of 50 ns as a function of time. As shown in Fig. 5D, the number of H-bonds formed between KWEKPF and ACE were fluctuated within 8–13 at the beginning 0–50 ns MD simulation, while the number of hydrogen bonds formed by FDRPFL with ACE gradually decreases and stabilized at 5. while the H-bonds numbers formed by FDRPFL with ACE were fluctuated within 3–7 at the 0–50 ns MD simulation. In comparison, KWEKPF formed more hydrogen bonds with ACE than FDRPFL formed, which is consistent with the molecular docking results. RMSF values revealed fluctuations in root mean square of receptor residues in MD simulations and can be used to assess complex stability (Pan et al., 2020). As shown in Fig. 5E, the overall fluctuation of amino acid residues in the ACE–peptide complex was significantly stronger than ACE. Hence, this suggested that FDRPFL and KWEKPF could bind to ACE to establish a protein–peptide complex. From RMSF results, it was found that six peaks of the protein-peptide complex had significantly higher RMSF values than ACE, and presumably ACEIPs formed hydrogen bonds and ionic interactions with ACE, thus resulting in larger fluctuations. For instance, FDRPFL may have formed hydrogen bonds or ionic interactions with amino acid residues Ile75, Ser129, Val298 and Pro299 in ACE, while KWEKPF may have formed hydrogen bonds with amino acid residues Arg228, Met296 and Leu439 in ACE.

#### Analysis of free-energy landscape maps

**Supplementary figure 3:**
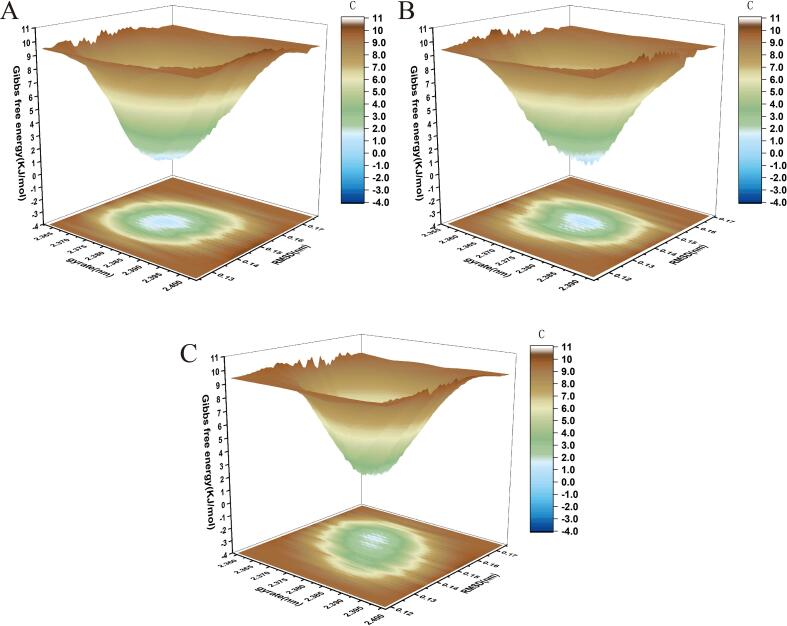


RMSD, Rg and free energy values of ACE–FDRPFL and ACE–KWEKPF complexes were used to construct free energy morphology maps to explore the conformational changes at different energy states, and the results are shown in Fig. S3. The three-dimensional and two-dimensional free energy landscape maps revealed that the free energy of the ACE–FDRPFL and ACE–KWEKPF complexes was lower than that of non-complexed ACE (Fig. S3C), indicating that the complexes had higher stability compared to non-complexed ACE. The complex ACE–FDRPFL had the lowest energy and the most stable structure when RMSD was within the range of 0.140–0.160 nm and Rg within the range of 2.375–2.385 nm (Fig. S3A). Similarly, the complex ACE–KWEKPF had the lowest energy and the most stable structure when RMSD was within the range of 0.135–0.145 nm and Rg within the range of 2.375–2.385 nm (Fig. S3B).

#### Determination of molecular mechanics generalized born surface area (MMPBSA)

The binding free energy between ACE–FDRPFL and ACE–KWEKPF was calculated using the MMPBSA method, and the results are shown in Table S4. Electrostatic and van der Waals energies were the main contributors to the affinity of ACE and both peptides FDRPFL and KWEKPF, which is consistent with the results of Song et al. (2023) The molecular docking results indicated that hydrogen bonds and ionic bonds were the main driving forces underlying the interaction between ACE and both FDRPFL and KWEKPF, suggesting that hydrogen bonds and ionic bonds played a key role in electrostatic energy. The total binding free energy of FDRPFL and KWEKPF when bound individually to ACE was −15.888 and −50.198 kcal/mol, respectively, which indicated that the two ACEIPs have binding affinity to the target protein ACE. In comparison, the binding energy of KWEKPF was lower than that of FDRPFL, indicating a higher binding rate to ACE. Interestingly, the ACE inhibitory activity of KWEKPF (IC_50_ = 0.75 ± 0.11 mg/mL) was lower than that of FDRPFL (IC_50_ = 0.67 ± 0.04 mg/mL), but the binding energy of KWEKPF was lower than that of FDRPFL. This may be due to the fact that ACE inhibition pattern of FDRPFL was non-competitive, and the binding of FDRPFL to the substrate-ACE complex was low, which resulted in a low binding energy to ACE. In contrast, the ACE inhibition pattern of KWEKPF was a mixed inhibition pattern, which indicated that KWEKPF could directly interact with both the active and non-active sites of ACE, resulting in a high binding energy (Paisansak et al., 2021). In the current study, molecular docking and MD simulation showed that FDRPFL and KWEKPF form stable and compact complexes with ACE by calculating RMSD, Rg, RMSF, and SASA values. Hydrogen bonds and ionic bonds were the main driving forces underlying the interaction between ACE and both FDRPFL and KWEKPF. Meanwhile, key amino acid residues of FDRPFL and KWEKPF interacting with ACE were identified. The above findings might provide guidance for insights into the interaction mechanisms between ACEIPs and ACE, and outline a potential strategy for the high-throughput screening of bioactive peptides.

## Conclusion

In the present study, whey obtained after the Rushan cheese production was used successfully to produce a whey powder and, subsequently, RCWH containing peptides of different sizes and sequences, which exerted ACE inhibitory activities. In addition, two novel ACE inhibitory peptides FDRPFL and KWEKPF were successfully screened from RCWH. *β*-turn and *β-*sheet motifs in the secondary structure of the two novel peptides were the main contributing factors to ACE inhibitory activity and stability. FDRPFL showed a non-competitive inhibition pattern, while KWEKPF showed a mixed inhibition pattern. Furthermore, molecular docking analysis and MD simulations showed that hydrogen bonds, ionic bonds and van der Waals forces enabled FDRPFL and KWEKPF to form a stable and compact complex with ACE. Although peptidomics and bioinformatics-based approaches are a viable and innovative approach to improve conventional experiments, it should be noted that validation of the *in-vivo* activity of the screened peptides is necessary. In subsequent studies, we intend to explore the blood pressure-lowering activity *in-vivo* and bioaccesibility of ACEIP. In conclusion, peptides isolated from RCWH exhibited good ACE inhibitory activity and may enable the high-value utilization of Rushan cheese by-products.

## CRediT authorship contribution statement

**Guangqiang Wei:** Conceptualization; Data curation; Formal analysis; Roles/Writing - original draft. **Teng Wang:** Data curation; Investigation; Methodology; Software; Visualization. **Yiyan Li:** Data curation; Formal analysis. **Rong He:** Supervision; Formal analysis. **Aixiang Huang:** Resources, Supervision. **Xuefeng Wang:** Resources, Project administration; Supervision; Writing - review & editing.

## Declaration of competing interest

The authors declare that they have no known competing financial interests or personal relationships that could have appeared to influence the work reported in this paper.

## Data Availability

No data was used for the research described in the article.

## References

[b0005] Abdelhedi O., Nasri R., Mora L., Jridi M., Toldra F., Nasri M. (2018). *In silico* analysis and molecular docking study of angiotensin i-converting enzyme inhibitory peptides from smooth-hound viscera protein hydrolysates fractionated by ultrafiltration. Food Chemistry.

[b0010] Bustamante S.Z., González J.G., Sforza S., Tedeschi T. (2021). Bioactivity and peptide profile of whey protein hydrolysates obtained from colombian double-cream cheese production and their products after gastrointestinal digestion. LWT - Food Science and Technology.

[b0015] Cushman D.W., Cheung S.H. (1971). Spectrophotometric assay and properties of the angiotensin-converting enzyme of rabbit lung. Biochemical Pharmacology.

[b0020] Cai S., Singh B.R. (1999). Identification of β-turn and random coil amide III infrared bands for secondary structure estimation of proteins. Biophysical Chemistry.

[b0025] Cheung L.K.Y., Aluko R.E., Cliff M.A., Li-Chan E.C.Y. (2015). Effects of exopeptidase treatment on antihypertensive activity and taste attributes of enzymatic whey protein hydrolysates. Journal of Functional Foods.

[b0030] Chen J., Ryu B., Zhang Y., Liang P., Li C., Qian Z. (2020). Comparison of an angiotensin-I-converting enzyme inhibitory peptide from tilapia (Oreochromis niloticus) with captopril: Inhibition kinetics, in vivo effect, simulated gastrointestinal digestion and a molecular docking study. Journal of the Science of Food and Agriculture.

[b0040] Coscueta E.R., Batista P., Gomes J.E., da Silva R., Pintado M.M. (2022). Screening of novel bioactive peptides from goat casein: *In silico* to *in vitro* validation. International Journal of Molecular Sciences.

[b0045] Cui Q., Duan Y.Q., Zhang M.J., Liang S.X., Sun Y.X., Guo M.Q. (2022). Peptide profiles and antioxidant capacity of extensive hydrolysates of milk protein concentrate. Journal of Dairy Science.

[b0050] Gobbetti M., Ferranti P., Smacchi E., Goffredi F., Addeo F. (2000). Production of angiotensin-I-converting-enzyme-inhibitory peptides in fermented milks started by *Lactobacillus delbrueckii subsp. bulgaricus* SS1 and *Lactococcus lactis subsp. cremoris* FT4. Applied and Environmental Microbiology.

[b0055] Guo Y.X., Jiang X.X., Xiong B.Y., Zhang T., Zeng X.Q., Pan D.D. (2018). Production and transepithelial transportation of angiotensin-I-converting enzyme (ACE)-inhibitory peptides from whey protein hydrolyzed by immobilized Lactobacillus helveticus proteinase. Journal Of Dairy Science.

[b0060] Hou C.J., Nie C.Q., Wang Y.Q., Ai L.Z., Xia Y.J., Wang G.Q. (2021). Rapid screening and verification of α-Lactalbumin-derived ACE inhibitory peptides. Food Science.

[b0065] Jin Y., Yu Y., Qi Y., Wang F., Yan J., Zou H. (2016). Peptide profiling and the bioactivity character of yogurt in the simulated gastrointestinal digestion. Journal of Proteomics.

[b0070] Li Y.S. (2021). Preparation of large yellow croaker ACE inhibitory peptide and its inhibitory Mechanism study. Nanchang University.

[b0075] Li X.Y., Feng C.S., Hong H., Zhang Y., Luo Z.G., Tan Y. (2022). Novel ACE inhibitory peptides derived from whey protein hydrolysates: Identification and molecular docking analysis. Food Bioscience.

[b0080] Li Y.Y., Fan Y.Z., Liu J.L., Meng Z.S., Huang A.X., Wang X.F. (2023). Identification, characterization and *in vitro* activity of hypoglycemic peptides in whey hydrolysates from rubing cheese by-product. Food Research International.

[b0085] Martini S., Conte A., Tagliazucchi D. (2020). Effect of ripening and *in vitro* digestion on the evolution and fate of bioactive peptides in parmigiano-reggiano cheese - sciencedirect. International Dairy Journal.

[b0090] Martini S., Solieri L., Tagliazucchi D. (2020). Peptidomics: New trends in food science. Current Opinion in Food Science.

[b0095] Mirzaei M., Mirdamadi S., Ehsani M.R., Aminlari M. (2018). Production of antioxidant and ACE-inhibitory peptides from *kluyveromyces marxianus* protein hydrolysates: Purification and molecular docking. Journal of Food and Drug Analysis.

[b0100] Mma, A., Rc, A., Lcp, A., Sps, B., & Akra, C. (2022). Characterization of ACE inhibitory and antioxidant peptides in yak and cow milk hard chhurpi cheese of the *sikkim himalayan region*. *Food Chemistry: X*, *13*, Article 10023.1 https://doi.org/10.1016/J.FOCHX.2022.100231. 10.1016/j.fochx.2022.100231 PMC9039942 35499015

[b0105] Mudgil P., Baby B., Ngohc Y.Y., Kamal H., Vijayan R., Gan C.Y. (2019). Molecular binding mechanism and identification of novel anti-hypertensive and anti-inflammatory bioactive peptides from camel milk protein hydrolysates. LWT-Food Science & Technology.

[b0110] Paisansak S., Sangtanoo P., Srimongkol P., Saisavoey T., Reamtong O., Karnchanatat A. (2021). Angiotensin-I converting enzyme inhibitory peptide derived from the shiitake mushroom (Lentinula edodes). Journal of Food Science and Technology-Mysore.

[b0115] Pan F., Li J., Zhao L., Tuersuntuoheti T., Mehmood A., Lin W. (2020). A molecular docking and molecular dynamics simulation study on the interaction between cyanidin-3-O-glucoside and major proteins in cow’s milk. Journal of Food Biochemistry.

[b0120] Pihlanto A., Virtanen T., Korhonen H. (2010). Angiotensin i converting enzyme (ACE) inhibitory activity and antihypertensive effect of fermented milk. International Dairy Journal.

[b0125] Sanjukta S., Padhi S., Sarkar P., Singh S.P., Rai A.K. (2021). Production, characterization and molecular docking of antioxidant peptides from peptidome of kinema fermented with *proteolytic bacillus spp*. Food Research International.

[b0130] Shao Y.Q. (2022). Preparation and properties of collagen ace inhibitory peptides from bone of eel. Zhejiang Ocean University.

[b0135] Shi Y., Wei G., Huang A. (2021). Simulated in vitro gastrointestinal digestion of traditional Chinese Rushan and Naizha cheese: Peptidome profiles and bioactivity elucidation. Food Research International.

[b0140] Shih Y.H., Chen F.A., Wang L.F., Hsu J.L. (2019). Discovery and study of novel antihypertensive peptides derived from cassia obtusifolia seeds. Journal of Agricultural and Food Chemistry.

[b0145] Song W.T., Fu J.X., Zeng Q., Lu H.Y., Wang J., Liu C.L. (2023). Improving ACE inhibitory activity of hazelnut peptide modified by plastein: Physicochemical properties and action mechanism. Food Chemistry.

[b0150] Tanzadehpanah H., Asoodeh A., Mahaki H., Mostajabodave Z., Chamani J., MojallalTabatabaei Z., Emtenani S. (2016). Bioactive and ACE binding properties of three synthetic peptides assessed by various spectroscopy techniques. Process Biochemistry.

[b0155] Tavares T.G., Malcata F.X. (2012). The Portuguese paradox: Why do some inhabitants of Portugal appear to live so long when their diet is based on whey cheese. Food Chemistry.

[b0160] Tu M., Wang C., Chen C., Zhang R., Liu H., Liu W., Du M. (2018). Identification of a novel ACE-inhibitory peptide from casein and evaluation of the inhibitory mechanisms. Food Chemistry.

[b0165] Tuly J.A., Zabed H.M., Nizami A.S., Hassan M.M., Azam S.M.R., Awasthi K.M., Ma H. (2022). Bioconversion of agro-food industrial wastes into value-added peptides by a bacillus sp. mutant through solid-state fermentation. Bioresource Technology.

[b0170] Wei G.Q., Wang D.D., Wang T., Shi Y.N., Huang A.X. (2022). Insights into *in vitro* digestion properties and peptide profiling of Chinese rubing PDO cheese prepared using different acidification technology. Food Research International.

[b0175] Wei G.Q., Zhao Q., Wang D.D., Fan Y.Z., Shi Y.N., Huang A.X. (2022). Novel ACE inhibitory, antioxidant and α-glucosidase inhibitory peptides identified from fermented rubing cheese through peptidomic and molecular docking. LWT - Food Science and Technology.

[b0180] Xia Y., Yu J., Xu W., Shuang Q. (2020). Purification and characterization of angiotensin-I-converting enzyme inhibitory peptides isolated from whey proteins of milk fermented with *Lactobacillus plantarum* QS670. Journal Of Dairy Science.

[b0185] Xie D., Du L., Lin H., Su E., Shen Y., Xie J., Wei D. (2022). *In vitro-in silico* screening strategy and mechanism of angiotensin i-converting enzyme inhibitory peptides from α-lactalbumin. LWT - Food Science and Technology.

[b0190] Yu Z., Liu B., Zhao W., Yin Y., Liu J., Feng C. (2012). Primary and secondary structure of novel ACE-inhibitory peptides from egg white protein. Food Chemistry.

[b0195] Yu Z., Guo H., Shiuan D., Xia C., Liu J. (2020). Interaction mechanism of egg white- derived ACE inhibitory peptide TNGIIR with ACE and its effect on the expression of ace and at1 receptor. Food Science and Human Wellness.

[b0200] Yu Z.P., Wang Y.X., Shuian D., Liu J.B., Zhao W.Z. (2023). Identification and molecular mechanism of novel immunomodulatory peptides from gelatin hydrolysates: Molecular docking, dynamic simulation, and cell experiments. Journal of Agricultural and Food Chemistry.

[b0205] Zhang B., Liu J., Wen H., Jiang F., Wang E., Zhang T. (2022). Structural requirements and interaction mechanisms of ACE inhibitory peptides: Molecular simulation and thermodynamics studies on LAPYK and its modified peptides. Food Science and Human Wellness.

[b0210] Zhang B.Y., Wang C., Xie Y.Y., Ren H.W., Fan W.G., Huang S.Y., Chen W. (2017). Optimization of preparation of Tibetan sheep placenta peptide by ultrasonic–assisted composite enzymatic hydrolysis. Science and Technology of Food Industry.

[b0215] Zhao Q., Wei G.Q., Li K.L., Duan S.H., Ye R., Huang A.X. (2022). Identification and molecular docking of novel α-glucosidase inhibitory peptides from hydrolysates of Binglangjiang buffalo casein. LWT- Food Science and Technology.

[b0220] Zhou M., Ren G.Y., Zhang B., Ma F.L., Fan J.L., Qiu Z.J. (2022). Screening and identification of a novel antidiabetic peptide from collagen hydrolysates of Chinese giant salamander skin: Network pharmacology, inhibition kinetics and protection of IR-HepG2 cells. Food Function.

