# Supplementary material for: Identification, structural characterization, and molecular dynamic simulation of ACE inhibitory peptides in whey hydrolysates from Chinese Rushan cheese by-product
Source: Food Chem X. 2024 Feb 10;21:101211. doi: 10.1016/j.fochx.2024.101211 (PMC10878854; doi:10.1016/j.fochx.2024.101211)
Supplement: Supplementary data 1 [file mmc1.doc]

**Table S1. Peptides identified from Rushan cheese whey hydrolysates (RCWH).**

| No. | Peptide sequence | No. | Peptide sequence |
| --- | --- | --- | --- |
| 1 | VYPFPGPIP | 49 | WMHQPH |
| 2 | GPVRGPFPI | 50 | VIESPPEIN |
| 3 | SKVLPVPQ**K** | 51 | **F**FSDKIA**K** |
| 4 | QEPVLGPV**R** | 52 | **K**TEIPTIN |
| 5 | QTPVVVPP**F** | 53 | MAIPPKKN |
| 6 | PFPGPIHNS | 54 | MAIPPKKN |
| 7 | QSKVLPVPQ | 55 | **F**MAIPPK**K** |
| 8 | SKVLPVPQ | 56 | MAIPPK**K** |
| 9 | PFPGPIPN | 57 | **F**MAIPP**K** |
| 10 | **K**VLPVPQ**K** | 58 | **K**YIPIQY |
| 11 | YPFPGPIP | 59 | **F**MAIPP**K** |
| 12 | VYPFPGPI | 60 | HPHPH**L** |
| 13 | GPVRGPFP | 61 | **K**ILDKVGIN |
| 14 | **Y**PFPGPIH | 62 | **K**FLDDDLT |
| 15 | PFPGPIHN | 63 | DKFLDDD**L** |
| 16 | FPKYPVEP | 64 | DKVGINYW |
| 17 | LQDKIHP**F** | 65 | DKVGINY |
| 18 | TPVVVPP**F** | 66 | **K**FLDDD**L** |
| 19 | EPVLGPV**R** | 67 | QINNKIW |
| 20 | MPFPKYPV | 68 | **K**FLDDD |
| 21 | HLPLPLLQ | 69 | **K**EPMIGVN |
| 22 | PKYPVEP**F** | 70 | QKEPMIGV |
| 23 | QPHQPLPP | 71 | YKVPQLE |
| 24 | NLHLPLP**L** | 72 | **K**YKVPQ**L** |
| 25 | PHQPLPPT | 73 | **K**EPMIGV |
| 26 | VPPFLQPE | 74 | **K**EPMIGV |
| 27 | MPFPKYPV | 75 | EQLLRL**K** |
| 28 | QEPVLGPV | 76 | YKVPQ**L** |
| 29 | AVPYPQR | 77 | **R**NAVPITPT |
| 30 | YPFPGPI | 78 | NPWDQV**K** |
| 31 | **K**EMPFP**K** | 79 | YQKFPQ |
| 32 | **K**AVPYPQ | 80 | **K**PTPEGDLE |
| 33 | **F**PGPIHN | 81 | IDTPGHVD**F** |
| 34 | GPVRGP**F** | 82 | DLGPITR**K** |
| 35 | PVVVPP**F** | 83 | NLHLPK**L** |
| 36 | MPFPKYP | 84 | NPKLPLS |
| 37 | QDKIHP**F** | 85 | DDFFH**R** |
| 38 | **F**PKYPVE | 86 | YDFYP**R** |
| 39 | MPFPKY**P** | 87 | **F**YLEP**R** |
| 40 | VYPFPGP | 88 | **F**DRPF**L** |
| 41 | PVRGPFP | 89 | HGRLPW |
| 42 | **R**GPFPII | 90 | HVPAPW |
| 43 | MHQPHQP | 91 | **K**WEKP**F** |
| 44 | SWMHQPH | 92 | **F**NRPF**L** |
| 45 | PFPKYPV | 93 | **K**PTPEGDLE |
| 46 | DKIHP**F** | 94 | VRTPEVDDE |
| 47 | **F**PKYPV | 95 | **L**KPTPEGDLE |
| 48 | IHPFAQ |  |  |
