# Supplementary material for: Identification, structural characterization, and molecular dynamic simulation of ACE inhibitory peptides in whey hydrolysates from Chinese Rushan cheese by-product
Source: Food Chem X. 2024 Feb 10;21:101211. doi: 10.1016/j.fochx.2024.101211 (PMC10878854; doi:10.1016/j.fochx.2024.101211)
Supplement: Supplementary data 2 [file mmc2.doc]

**Table S2. Bioactive peptides identified in Rushan cheese whey hydrolysates (RCWH).**

| No. | Peptide sequence | Peptide ranker  score | Potential bioactive | Protein |
| --- | --- | --- | --- | --- |
| 1 | YPFPGPI | 0.917487 | ACE inhibitor, Antioxidative, Anticancer | Beta-casein |
| 2 | YPFPGPIP | 0.899039 | ACE inhibitor, Antioxidative | Beta-casein |
| 3 | PFPGPIPN | 0.890369 | ACE inhibitor | Beta-casein |
| 4 | VYPFPGPI | 0.849683 | Antihypertensive | Beta-casein |
| 5 | VYPFPGPIP | 0.810918 | Antihypertensive | Beta-casein |
| 6 | DKIHPF | 0.686943 | ACE inhibitor | Beta-casein |
| 7 | AVPYPQR | 0.567162 | ACE inhibitor, Antimicrobial, Antioxidative | Beta-casein |
| 8 | MAIPPKK | 0.506137 | ACE inhibitor | Kappa-casein |
| 9 | DKVGINYW | 0.387484 | ACE inhibitor | Alpha-lactalbumin |
| 10 | LKPTPEGDLE | 0.193857 | Dipeptidyl peptidase IV inhibitor | Beta-lactoglobulin |
| 11 | SKVLPVPQ | 0.180961 | ACE inhibitor | Beta-casein |
| 12 | KVLPVPQK | 0.159789 | ACE inhibitor, Antioxidative | Beta-casein |
| 13 | DKVGINY | 0.13778 | ACE inhibitor | Alpha-lactalbumin |
