# Supplementary material for: Identification, structural characterization, and molecular dynamic simulation of ACE inhibitory peptides in whey hydrolysates from Chinese Rushan cheese by-product
Source: Food Chem X. 2024 Feb 10;21:101211. doi: 10.1016/j.fochx.2024.101211 (PMC10878854; doi:10.1016/j.fochx.2024.101211)
Supplement: Supplementary data 3 [file mmc3.doc]

**Table S3.** Estimated binding energy and chemical interactions for effective conformations as obtained by molecular docking analysis of synthetic peptides FDRPFL and KWEKPF from Rushan cheese whey hydrolysates at the angiotensin-I-converting enzyme (ACE) pocket.

| No. | Sequence | Binding energy/ kcal/mol | Residues formed hydrogen bonds with the ligand | Peptide binding  residues | Number of hydrogen bonds | Number of hydrophobic amino acid residues | Number of  ionic bond |
| --- | --- | --- | --- | --- | --- | --- | --- |
| P1 | FDRPFL | -9.60 | Arg402, Glu124 | R1, L1 | 2 | 16 | 7 |
| P2 | KWEKPF | -7.40 | Glu411, His387, His383, His353,  Ala356, Glu403 | K5, W1 | 6 | 16 | 0 |
