# Supplementary material for: Identification, structural characterization, and molecular dynamic simulation of ACE inhibitory peptides in whey hydrolysates from Chinese Rushan cheese by-product
Source: Food Chem X. 2024 Feb 10;21:101211. doi: 10.1016/j.fochx.2024.101211 (PMC10878854; doi:10.1016/j.fochx.2024.101211)
Supplement: Supplementary data 4 [file mmc4.doc]

**Table S4.** Binding free energy values of peptides FDRPFL and KWEKPF complexed separately with angiotensin-I-converting enzyme (ACE) calculated using the molecular mechanics generalized born surface area (MMPBSA) method.

| Energy | ACE-FDRPFL | ACE-KWEKPF |
| --- | --- | --- |
| Van der Waals Energy(KJ/mol) | -287.120 | -287.968 |
| Electrostatic energy (kJ/mol) | -218.506 | -203.069 |
| Polar solvation energy (KJ/mol) | 488.766 | 412.037 |
| Nonpolar solvation energy(KJ/mol) | -36.105 | -21.802 |
| Total binding energy(KJ/mol) | -52.965 | -100.803 |
| Total binding free energy(KJ/mol) | -15.888 | -50.198 |
